# Cost-Effectiveness Analysis: Risk Stratification of Nonalcoholic Fatty Liver Disease (NAFLD) by the Primary Care Physician Using the NAFLD Fibrosis Score

**DOI:** 10.1371/journal.pone.0147237

**Published:** 2016-02-23

**Authors:** Elliot B. Tapper, M. G. Myriam Hunink, Nezam H. Afdhal, Michelle Lai, Neil Sengupta

**Affiliations:** 1 Division of Gastroenterology/Hepatology, Department of Medicine, Beth Israel Deaconess Medical Center, Harvard Medical School, Boston, MA, 02215, United States of America; 2 Dept of Radiology, Dept of Epidemiology, Erasmus University Medical Center, Rotterdam, the Netherlands; 3 Center for Health Decision Sciences, Harvard T.H. Chan School of Public Health, Boston, United States of America; 4 Division of Gastroenterology/Hepatology/Nutrition, Department of Medicine, University of Chicago, Chicago, Illinois; RWTH Aachen, GERMANY

## Abstract

**Background:**

The complications of Nonalcoholic Fatty Liver Disease (NAFLD) are dependent on the presence of advanced fibrosis. Given the high prevalence of NAFLD in the US, the optimal evaluation of NAFLD likely involves triage by a primary care physician (PCP) with advanced disease managed by gastroenterologists.

**Methods:**

We compared the cost-effectiveness of fibrosis risk-assessment strategies in a cohort of 10,000 simulated American patients with NAFLD performed in either PCP or referral clinics using a decision analytical microsimulation state-transition model. The strategies included use of vibration-controlled transient elastography (VCTE), the NAFLD fibrosis score (NFS), combination testing with NFS and VCTE, and liver biopsy (usual care by a specialist only). NFS and VCTE performance was obtained from a prospective cohort of 164 patients with NAFLD. Outcomes included cost per quality adjusted life year (QALY) and correct classification of fibrosis.

**Results:**

Risk-stratification by the PCP using the NFS alone costs $5,985 per QALY while usual care costs $7,229/QALY. In the microsimulation, at a willingness-to-pay threshold of $100,000, the NFS alone in PCP clinic was the most cost-effective strategy in 94.2% of samples, followed by combination NFS/VCTE in the PCP clinic (5.6%) and usual care in 0.2%. The NFS based strategies yield the best biopsy-correct classification ratios (3.5) while the NFS/VCTE and usual care strategies yield more correct-classifications of advanced fibrosis at the cost of 3 and 37 additional biopsies per classification.

**Conclusion:**

Risk-stratification of patients with NAFLD primary care clinic is a cost-effective strategy that should be formally explored in clinical practice.

## Introduction

Nonalcoholic fatty liver disease (NALFD) is increasingly common, afflicting no less than 1 in 5 people living in Western nations. [1–3] In America, the respective prevalence of steatosis and steatohepatitis is 46% and 12% and rising[4,5] and outpatient visits for the primary purpose of NAFLD management have doubled of late.[3,6] The contemporary management of patients with NAFLD is defined by two key features. First, there is a lack of a definite therapeutic modality beyond lifestyle modification. Second, advanced fibrosis is the only significant predictor of long-term complications and excess mortality. [7,8] Accordingly, a major focus of clinical care for patients with NAFLD should be the determination of those at highest risk for the complications of advanced liver disease.[1,4]

The optimal strategy for the management of NAFLD would benefit from a multidisciplinary approach to prioritize patients for specialists referral. Patients with advanced fibrosis must be discovered and managed appropriately by providing intensified therapy and screening procedures for hepatocellular carcinoma and portal hypertension. Liver biopsy, while still considered a gold-standard, is not preferred by patients and is too costly and risky to be applied to a highly prevalent condition.[9] We recently showed that two strategies for non-invasive assessment for advanced fibrosis were cost-effective compared to usual care (biopsy).[10] In this model, we used the NAFLD Fibrosis Score (NFS)[11]–a risk prediction algorithm based on simple blood tests and body mass index (BMI)–and the combination of the NFS with vibration-controlled elastography (VCTE)[12]–a Food and Drug Administration approved ultrasound-based technique for fibrosis risk assessment. At the same time, universal referral to a gastroenterologist for such a common disease is untenable. Ideally, primary care physicians would recognize and manage early disease while gastroenterologists are referred patients with advanced liver disease.

Increasingly, reports are identifying the feasibility of screening for advanced liver disease in the primary care setting. [13,14] Doycheva and colleagues used magnetic resonance imaging (MRI) techniques in a cohort of consecutive diabetic patients enrolled from primary care clinics in San Diego.[14] They identified that 7.1% of patients were at risk for advanced fibrosis based on liver stiffness measurements. Kwok et al screened 1918 patients from Hong Kong with diabetes for the presence of NAFLD and advanced fibrosis with vibration-controlled elastography (VCTE) based techniques, 17.1% of whom were identified as high risk for advanced fibrosis.[13] While only 94 of these patients underwent biopsy, half of them had F3-F4 fibrosis.[13,14] The cost-effectiveness of this strategy is unknown. Indeed, the cost of adopting a new technology in the primary care setting is unclear. Furthermore, non-invasive tests can yield both false-positive and false-negative results, the long-term implications of which are unknown.

Herein, this study compares the cost-effectiveness of multiple strategies for the evaluation of patients with NAFLD in the primary care clinic or the referral setting using the NFS, VCTE, and their combination to the current standard of percutaneous liver biopsy.

## Methods

We developed a probabilistic decision analytical microsimulation state-transition model[15] using dedicated software (DATA 3.5, TreeAge, Williamstown, MA). The analysis was performed according to published guidelines.[16,17] We simulated a cohort of 10,000 50-year old patients evaluated in the primary care clinic for elevated liver enzymes (>40 IU/L) and liver ultrasound consistent with steatosis due to NAFLD. This model recapitulated the natural history of NAFLD over 30 years All patients had a negative evaluation for excessive alcohol intake, hepatitis C, hepatitis B, drug-induced liver injury or hemochromatosis. We assume that patients with decompensated cirrhosis have been diagnosed clinically and managed accordingly. We assume that prior to entry into the model each patient was observed during a trial of lifestyle modification with persistent elevations in their liver enzyme elevation when reassessed after a six-month period. All patients have NAFLD in this model with an unknown fibrosis stage. The costs and benefits considered from the societal perspective.

**Fig 1:**
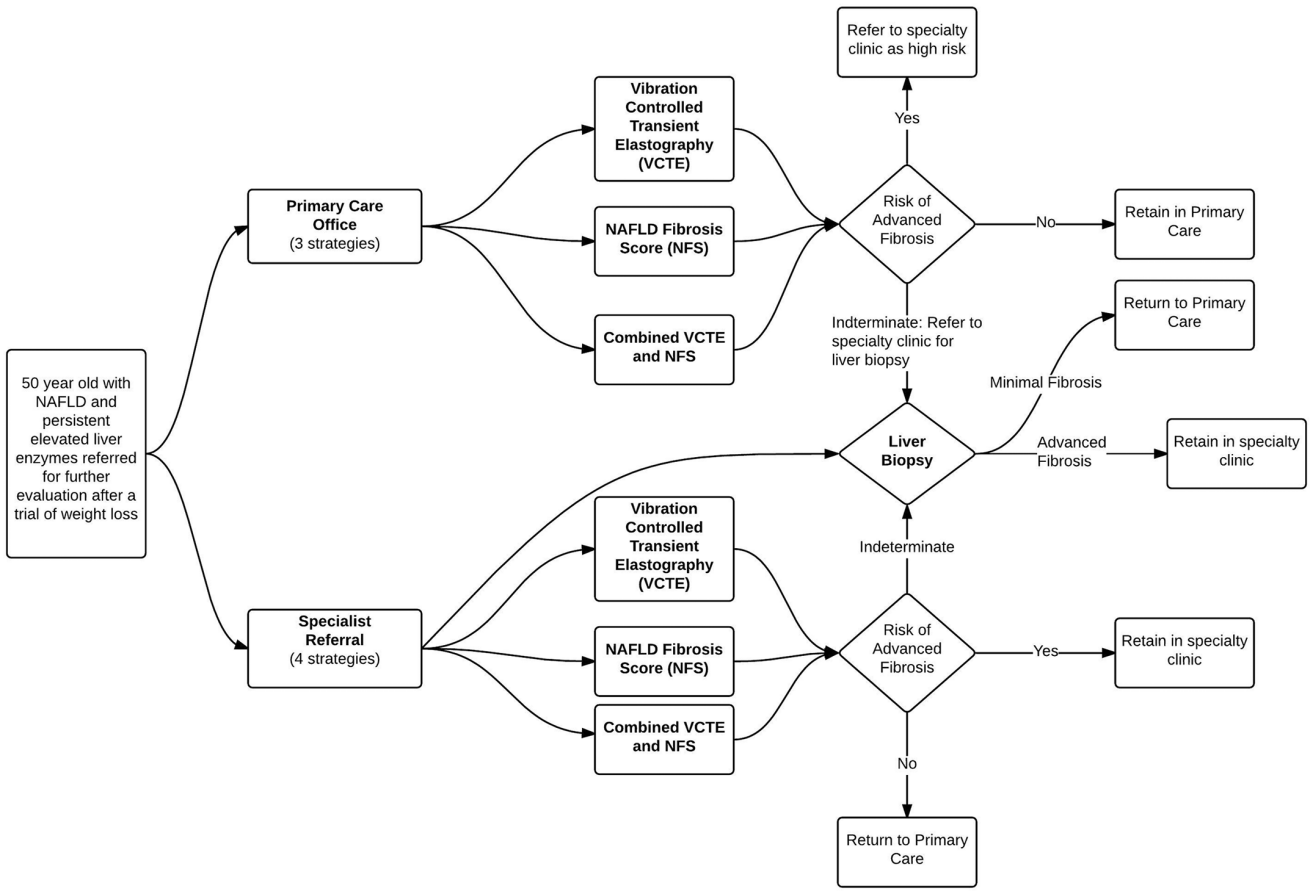
Flowchart of Patient Evaluation By Strategy.

We compared the following seven evaluation strategies: 6 noninvasive strategies (NFS alone, VCTE alone, combined NFS and VCTE; each in the PCP and Liver clinic), and liver biopsy in the liver clinic (Fig 1).[4,12] Each of the non-invasive strategies assigns patients into one of three categories: low, indeterminate and high risk of advanced fibrosis. The clinical decisions associated with each non-invasive strategy included referral to or retention in liver clinic for advanced fibrosis and referral back to or retention in the PCP office for early stage fibrosis. Consistent with expert recommendations, only the patients with indeterminate non-invasive results are offered a liver biopsy.[12,18] Test characteristics and clinical decision criteria are detailed in Table 1.

**Table 1:** Estimates of Test Performance and Disease Prevalence for the Model.

Model Variable	Estimate (%)	Reference
Disease Stage Prevalence		
Cirrhosis	0.06	[26], local data
Advanced (METAVIR 3) Fibrosis	0.11	[26], local data
NASH without Advanced Fibrosis	0.37	[26], local data
Simple Steatosis	0.47	[26], local data
**Liver Biopsy Sensitivity and Specificity**		
Advanced Fibrosis	0.85 and 0.89	[27]
NASH	0.88 and 0.74	[27]
**VCTE**		
Sensitivity and Specificity for Advanced Fibrosis	0.95 and 0.77	Local data
Indeterminate results—AdvancedFibrosis	1.0	Local data
Indeterminate results—NASH	0.21	Local data
Indeterminate results—Simple Steatosis	0.11	Local data
**NFS**		
Sensitivity and Specificity for Advanced Fibrosis	0.21–0.43 and 0.96	[11], local data
Indeterminate results—Advanced Fibrosis	0.57–0.62	[11], local data
Indeterminate results—NASH	0.25–0.46	[11], local data
Indeterminate results—Simple Steatosis	0.06–0.25	[11], local data
**VCTE and NFS**		
Sensitivity and Specificity for Advanced Fibrosis	0.19–0.83 and 0.99–1.0	[31], local data
Indeterminate results—AdvancedFibrosis	0.48–0.81	[31], local data
Indeterminate results—NASH	0.46–0.55	[11], local data
Indeterminate results—Simple Steatosis	0.06–0.37	[11], local data

The values presented are for the base-case estimate. In the microsimulation model, each value is given a wide distribution that is sampled 10, 000 times.

NASH = Nonalcoholic Steatohepatitis, NFS = NAFLD Fibrosis Score, VCTE = Vibration Controlled Transient Elastography

**Fig 2:**
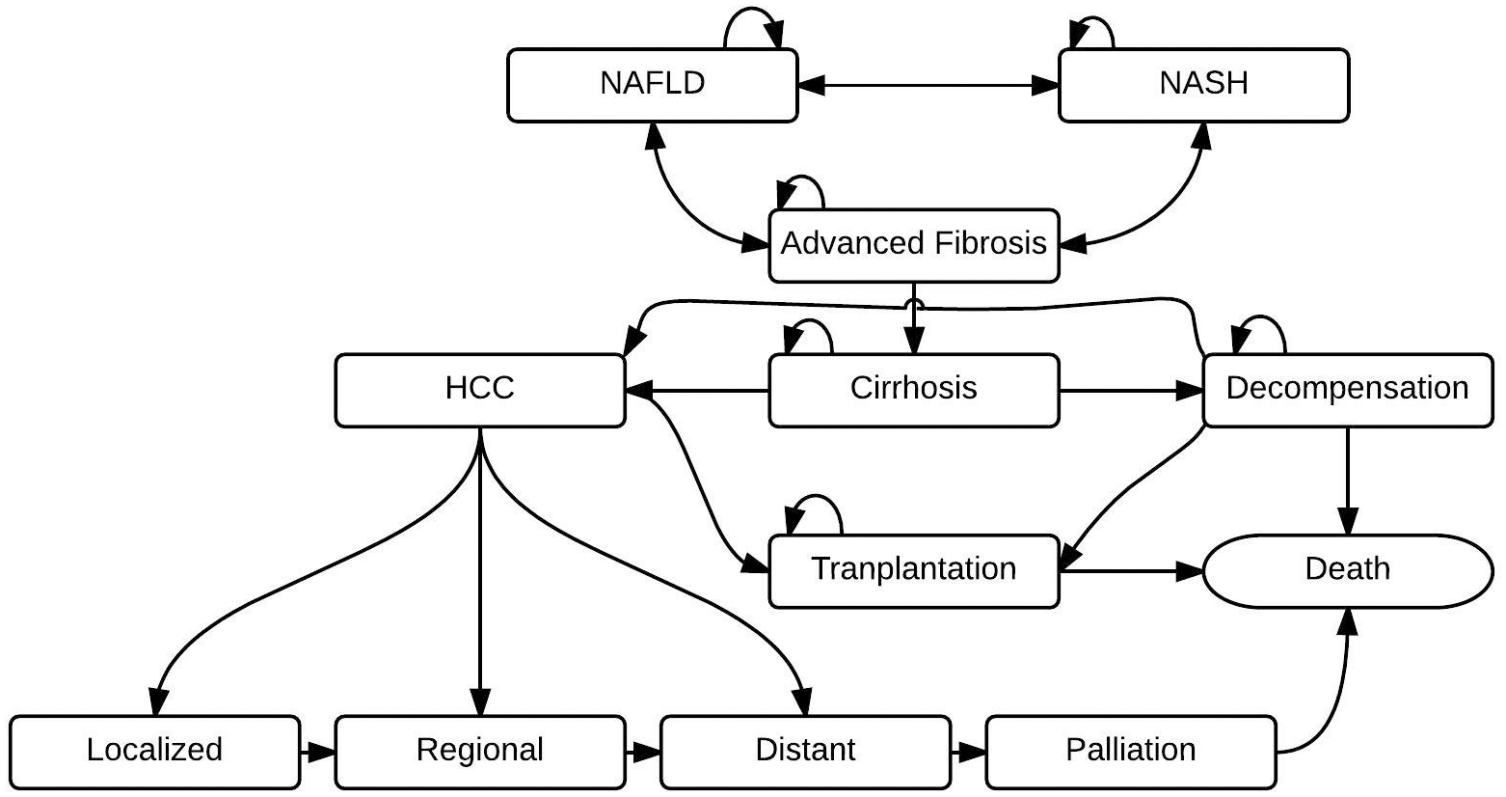
A Simplified Depiction of the Microsimulation State-Transition Model. HCC = Hepatocellular carcinoma; NAFLD = Nonalcoholic Fatty Liver Disease; NASH = Nonalcoholic Steatohepatitis.

Each patient enters into the microsimulation state-transition model to simulate the clinical and economic outcomes associated with each clinical decision (Fig 2). Patients stratified as high risk for advanced liver disease (true and false positives) are followed closely by specialists and incur increased costs and decreased utilities associated with care for advanced liver disease (e.g. specialist visits, screening tests). Patients classified as low-risk are followed by their primary care physicians. Regardless of stratification, all patients follow the clinical trajectory of their true disease-stage.

### Ethics

This study does not include human subjects research.

### Definition of Terms

The goal of this analysis is to model two outcomes simultaneously based on the generation of discounted costs (2014 US dollars) and discounted quality adjusted life years (QALY) that accrue to our cohort over time. Each model input which governs a patient's natural history is a probability and beyond that there is a distribution of values (uncertainty) for most clinical variables—e.g. confidence intervals or ranges of values in the literature. For this reason, the model is called a microsimulation—which follows the stochastic movements of individual subjects through the chances of developing clinical outcomes—and a probabilistic analytic model which analyzes 10,000 random samples within each parameter’s distribution. Accordingly, this method repeats the analysis with 10,000 permutations of the variable inputs, thereby performing sensitivity analyses for each variable within its distribution. The end result is the probability of cost-effectiveness for a given strategy in the overall set of simulations. The relative cost-effectiveness of the strategies, in turn, are adjudicated with reference to a society’s willingness-to-pay (WTP) threshold. The WTP threshold is the amount of money per person that society is willing to pay to adopt a new clinical strategy for an additional QALY over the current acceptable strategy. Finally, we calculated a metric called the population ‘expected value of perfect information’ (EVPI). The population EVPI is a reflection of the benefit derived from further research and is therefore a measure of the uncertainty in this analysis.

### State-Transition probabilities

Details regarding the transition probabilities in the microsimulation model are described in detail within the supplementary methods and supplementary table 1 (Table A in S1 Appendix). In brief, the natural history of NAFLD was simulated utilizing published data. Patients with NASH could regress to NAFLD. Similarly, patients with advanced fibrosis could regress to NASH without advanced fibrosis. The rate of regression with lifestyle changes was derived from longitudinal cohort studies utilizing sequential biopsies and subsequently converted to probabilities. [19,20] In our model, patients with cirrhosis could not regress to an earlier stage of disease. The annual rate of fibrosis progression was abstracted from a meta-analysis by Singh et al: 0.07 fibrosis stages (95% CI, 0.02–0.11) for simple steatosis and 0.14 fibrosis stages (95% CI, 0.07–0.21) for those with NASH.[21]

Treatments and screening tests could modify the natural history. Both lifestyle interventions and vitamin E are associated with improvement of histological inflammation,[19,22,23] the magnitude of which was derived from a randomized control trial comparing both modalities.[19] In accordance with published guidelines, we restricted vitamin E to patients with biopsy-proven NASH.[4,19] As vitamin E does not lead to regression of advanced fibrosis,[19] these patients with were not candidates for therapy. Patients who were falsely 'upstaged' by their risk stratification test are therefore not offered vitamin E. Adherence to either lifestyle interventions or vitamin E therapy is assumed to be 50% based on estimates from the World Health Organization.[24] Patients with advanced fibrosis are screened for hepatocellular carcinoma (HCC). In accordance with a recent meta-analysis, patients who were correctly identified as having advanced fibrosis and were followed by a gastroenterologists experienced increased likelihood of early stage HCC detection (odds ratio (OR) 2.08 95% CI (1.80–2.37) and curative treatment (OR 2.24 95% CI (1.99–2.52)).[25] The costs of care and utilities associated with each state and strategy are detailed in the supplementary methods (Tables B-C in S1 Appendix).

### Disease-Stage prevalence

The population assessed in this model reflects patients who would otherwise be referred for consultation after a preliminary evaluation for elevated liver function tests in the setting of NAFLD. All patients have NAFLD. As previously published we derived the relative proportion of patients with simple steatosis, NASH without advanced fibrosis (METAVIR stage 0–2 fibrosis), advanced fibrosis (METAVIR stage 3 fibrosis) and cirrhosis from a cohort of 431 patients seen at our referral clinic from 1996–2013.[26] The combined proportion from these cohorts with simple steatosis, NASH without advanced fibrosis, advanced fibrosis, and cirrhosis are, respectively, 46.6% (201), 36.7% (158), 11.1% (48), and 5.6% (24).

### Strategy Characteristics

#### Liver biopsy

Liver biopsy is also susceptible to false positives and false negatives in the evaluation of NAFLD.[27] These values are described in Table 1. Further details are available in the supplementary methods (S1 Appendix). As the test characteristics of biopsy following a non-invasive risk-stratification are unknown, we assumed perfect biopsy performance in the non-invasive strategies.

#### VCTE

VCTE generates a discrete liver stiffness measurement. Different studies utilize different cutoffs for advanced fibrosis and cirrhosis. [28–30], The test characteristics are those from our center as published elsewhere.[10] We determined that a liver stiffness of 9.9 kPa was an optimal cutoff for advanced fibrosis (F3-F4). The range of indeterminate values have been suggested by expert opinion but have not been formally studied.[18] For our purposes, we used an indeterminate range of 20%, meaning that all patients with liver stiffness measurements between 7 and 9.8 kPa were considered indeterminate for advanced fibrosis. The technical details pertaining to the performance of VCTE and the statistical interpretation of the optimal cutoffs are presented in the supplementary methods (S1 Appendix).

#### NFS

The NAFLD Fibrosis Score is a readily available online calculator to risk-stratify patients with NAFLD for the presence of advanced liver disease. The validated test characteristics of the NFS strategy were derived from two sources: the landmark study by Angulo and colleagues[11] and our cohort. Similar to VCTE, indeterminate results are those with high-negative values. These values are summarized in Table 1. Further information is available in the supplementary methods (S1 Appendix).

#### NFS and VCTE

The combination of NFS and VCTE with liver biopsy for indeterminate results has been evaluated in a prospective study of Italian patients.[31] We applied this strategy to our cohort and the resultant test characteristics are described in Table 1. For the purpose of the model we used a distribution based on values derived from the paper by Petta et al as well as our cohort.[31] The presence or absence of advanced fibrosis is defined by concordance between the tests; indeterminate results from the combination strategy can indicate either test discordance (positive and negative) or indeterminate results from either test. If VCTE is unfeasible, then clinical decisions follow the strategy defined by NFS alone. See the supplementary methods for the determination of the proportion of unfeasible VCTE exams (S1 Appendix).

#### Data Analysis

The model assumed an annual cycle length and terminated after 30 years. All costs, life-years and utilities were discounted at a rate of 3%. Half-cycle correction was performed. All costs were inflated to 2014 values and converted to American dollars. One-way sensitivity analyses were performed for all variables and those which modified the outcome of the model were presented in the results.

The primary outcome is derived from probabilistic sensitivity analyses which were performed using Monte Carlo microsimulation of 10,000 patients with 10,000 samples taken from the input parameter distributions. We chose a conventional WTP threshold of $100,000.[32] We determined the expected value of perfect information (EVPI) as described in the supplemental methods (S1 Appendix).

The secondary outcome is correct classifications of advanced fibrosis per biopsy performed. In this model, we re-ran our microsimulation as a simple decision tree to determine the number of biopsies needed (e.g. cost) and the proportion of patients with correct classification of advanced fibrosis (i.e. effectiveness). This model does not assess differences between management in the PCP or referral clinic and instead focuses strictly on the performance of NFS, VCTE, NFS combined with VCTE and usual care (biopsy), penalizing poor sensitivity. It is also a microsimulation of 10,000 patients with 10,000 samples taken from the input parameter distributions pertinent to stratification test characteristics.

## Results

Compared to the standard care of performing a liver biopsy, all noninvasive strategies are cost-effective (Table 2). Strategies involving triage by the primary care physician (PCP) are less costly than those requiring initial referral to a gastroenterologist. Whereas a strategy employing the NFS alone by the PCP is cost-saving compared to usual care (referral for liver biopsy) by $13,585 per person, it is also cost-saving compared to initial referral where the evaluation is based on the NFS (by $2,118 per person). The NFS alone performed in the PCP’s office is a ‘dominant’ strategy with both cost-savings and increased effectiveness. The cost per QALY of the NFS is $5,985 in the PCP office and $6,138 after referral while usual care with liver biopsy costs $7,229. A cost-acceptability curve was generated to evaluate the cost-effectiveness of all strategies in a probabilistic sensitivity analysis. At a willingness-to-pay (WTP) threshold of $100,000 per QALY, the NFS alone strategy in the PCP office was dominant in 94.2% of samples, the NFS/VCTE strategy in the PCP office was dominant in 5.6% and usual care in 0.2%.

**Table 2:** The Cost and Effect of Non-invasive Evaluations of Nonalcoholic Fatty Liver Disease (NAFLD) Compared to Usual Care (Liver Biopsy) in the Primary Care and Referral Setting.

Strategy	Cost ($)	Effect (QALY)	Incremental Cost ($)	Incremental Effect (QALY)
Usual Care	98,815	13.67	+13,585	-0.57
VCTE(after referral)	97,881	13.74	+12,651	-0.50
VCTE(PCP office)	96,478	13.74	+11,248	-0.50
NFS/VCTE (after referral)	96,334	13.97	+11,104	-0.27
NFS/VCTE(PCP office)	94,942	13.97	+9,711	-0.27
NFS(after referral)	87,349	14.23	+2,118	-0.01
NFS(PCP office)	85,231	14.24		

Incremental costs and QALY reference a common baseline (usual care)

NFS = NAFLD Fibrosis Score, QALY = quality adjusted life-years, VCTE = Vibration Controlled Transient Elastography

While the non-invasive strategies are expected to be cost-saving, there is uncertainty in these results. The population-based EVPI is moderate, demonstrating that there is benefit to be derived from further research. At WTP of $100,000 per QALY, the EVPI is $142 Million.

The performance of each strategy relative to the number of correct diagnoses provided is detailed in Table 3. The NFS based strategies yield the best biopsy-to-correct classification ratios (3.5). Both usual care and the NFS/VCTE strategy yield more correct classifications. However, for each additional correct classification of advanced fibrosis related to NFS based strategies, the NFS/VCTE costs 3 additional biopsies and usual care costs 37. One-way sensitivity analyses were performed to explore these tradeoffs. The rate of VCTE failure before which it leads to fewer biopsies and higher correct classification rates than NFS based strategies is 17.5%. With current test characteristics, NFS leads to fewer biopsies than VCTE based strategies up to a 59% prevalence of advanced fibrosis.

**Table 3:** Strategy Performance in a cohort of 10,000 patients.

Strategy	Number of biopsies	Correct Classification of Advanced Fibrosis	Incremental Biopsies	Incremental Correct Classifications	Incremental Biopsy to Correct Classification Ratio
Usual Care	10,000	9,400	+7,357	+200	37
VCTE	3,485	8,400	+815	-800	*
NFS/VCTE	3,507	9,500	+864	+300	3
NFS	2,643	9,200			

NFS = Nonalcoholic Fatty Liver Disease Fibrosis Score, VCTE = Vibration Controlled Transient Elastography

*—negative incremental ratios are not calculated

## Discussion

The non-invasive evaluation of NAFLD using the NFS with or without VCTE is a cost-effective strategy for the patient-centered risk stratification of an increasingly common disease.[10] In this study of seven strategies comparing evaluation by primary care physicians (PCPs) and gastroenterologists, the use of NFS alone by the PCP was the most cost-effective strategy followed by the combination of NFS with VCTE (also by the PCP). These data demonstrate that tremendous costs are saved when a model of NAFLD care is applied where primary care physicians would recognize and manage early disease, referring to gastroenterologists the patients with advanced liver disease.

Our study adds to and extends the prior literature on the evaluation and management of NAFLD/NASH in two principle ways. First, we previously showed that the use of non-invasive indices was cost-effective in the referral setting.[10] This model extends that work by claiming additional cost-savings when PCPs participate in the risk-stratification of NAFLD. The benefits of widespread risk-stratification in the primary care setting are substantial for two major reasons. First, recent studies have demonstrated that when PCPs screen patients at risk for NAFLD—including those with normal liver enzymes[14]–the prevalence of advanced liver disease is high. Second, late referrals, which make up 15–80% of referrals in prior studies, are associated with suboptimal outcomes,[33] particularly as it relates to the lack of screening for HCC. Furthermore, up to 20% of patients referred to a specialist will not attend their visit.[34] While this model requires that PCPs accurately identify patients with NAFLD among those presenting with elevated liver enzymes, several studies have shown that PCPs are comfortable routinely excluding other causes of liver disease.[35–37]

Second, these data shed light on the reasons for the NFS’ robust performance. The NFS was developed by Angulo as a means to avoid liver biopsy except for those patients with indeterminate results.[11] VCTE can accomplish the same goals.[38] However, while VCTE offers an excellent negative predictive value, it is susceptible to false-positives, particularly owing to the contribution of inflammation and steatosis to liver stiffness.[39,40] These data demonstrate that by avoiding over-staging, the NFS and NFS/VCTE strategies are the most likely to identify high risk patients while minimizing false-positives and the need for biopsies. Unlike for patients with hepatitis C where over-staging may lead to unforeseen benefits such as approval for antiviral therapy, there are limited benefits for a patient with uncomplicated NAFLD being misclassified as having advanced fibrosis. While evaluation by a specialist and screening for liver complications has measurable health benefits, it is also costly and associated with the minor decrements in quality of life associated with frequent medical appointments. As long as the available data supports a generally benign natural history for patients with NAFLD, cost-effectiveness will be achieved by cost-containing strategies (i.e. NFS).

This study must be evaluated within the context of the study design. First, this model did not assess the benefits of controlled attenuation parameter (CAP) for VCTE which could identify patients with steatosis.[41] Nor did we evaluate MRI which can combine effective evaluations for steatosis and fibrosis in the same examination.[14] Both techniques, not yet approved for clinical use, are likely promising and should be explored with further clinical data before cost-effectiveness analysis. Second, data regarding the natural history of NAFLD is limited, particularly for patients with normal liver enzymes, and therefore multiple base-case estimates of the disease-stage prevalence, response to therapy and the performance of non-invasive diagnostics in patients without elevated liver enzymes were not possible. Accordingly, it is unclear whether these data are generalizable for the screened population. Third, this model should be contrasted with our prior work in several ways. First, the rate of fibrosis stage progression has received greater clarity owing to a recent meta-analysis by Singh and colleagues which now includes the possibility of NAFLD without NASH progressing directly to advanced fibrosis.[21] Second, in view of recent data on the benefit of hepatocellular cancer screening, we modeled a survival benefit for correct classification of advanced fibrosis vis-à-vis earlier stage diagnosis.[25] Third, we used our own experience with VCTE in the US to determine the rate of failed examinations which is 3 times the prior published estimate.[10] Fourth, we modelled the performance of NFS and VCTE in the same cohort instead of the prior dependence on published data. Taken together, these modifications result in incremental but significant improvements in a model which strives to recapitulate the natural history of NAFLD after a one-time risk stratification.

In conclusion, both the NFS and the combination of NFS with VCTE are biopsy-sparing, cost-effective tools in the evaluation and management of NAFLD in the primary care setting.

## Supporting Information

S1 Appendix Supplementary Methods. **Table A**: Transition Probabilities. **Tables B-C**: Model Costs and Health-state Utilities.
(DOCX)

## Abbreviations

NAFLD: Nonalcoholic fatty liver disease
NASH: Nonalcoholic steatohepatitis
NFS: NAFLD fibrosis score
VCTE: vibration controlled transient elastography
WTP: willingness-to-pay
EVPI: expected value of perfect information
QALY: quality adjusted life-years
HCC: Hepatocellular carcinoma
