# Supplementary material for: Cost-Effectiveness Analysis: Risk Stratification of Nonalcoholic Fatty Liver Disease (NAFLD) by the Primary Care Physician Using the NAFLD Fibrosis Score
Source: PLoS One. 2016 Feb 23;11(2):e0147237. doi: 10.1371/journal.pone.0147237 (PMC4764354; doi:10.1371/journal.pone.0147237)
Supplement: S1 Appendix — Table A: Transition Probabilities. Tables B-C: Model Costs and Health-state Utilities. (DOCX) [file pone.0147237.s001.docx]

**S1Appendix: Supplementary Methods**

Vibration Controlled Transient Elastography (VCTE)

Obesity can reduce the reliability of VCTE defined by the ability to obtain a valid result as well as a set of 10 valid measurements with an interquartile range < 30% of the median liver stiffness measurement value . We studied the success of VCTE examinations in a prospective cohort of 164 patients. An unreliable VCTE result was obtained in 26.8% of examinations. An unreliable result does not, in and of itself, imply increased risk of advanced fibrosis.[[1](#_ENREF_1)] As such, patients with failed VCTE are evaluated by either liver biopsy or NFS depending on the strategy arm without modification of their pretest probabilities. These patients are assessed by liver biopsy in our model. The cost of each VCTE examination is $100USD (2014), which includes training and startup costs. [[2](#_ENREF_2)]

**NAFLD Fibrosis Score (NFS)**

It is assumed that given the prior evaluation received by the patients in our model, the data needed for NFS is available at the time of clinical evaluation. These data include age, body mass index, diabetes, AST, ALT, platelet count and albumin. We employed the 0.676 cut-off to optimize NFS test characteristics given that the prevalence of advanced fibrosis in our sample exceeds 15%.[[3](#_ENREF_3)] The rate of low, indeterminate and high risk results was derived from a prospective study of 164 patients with NAFLD at our center as well as the original NFS manuscript.[[3](#_ENREF_3)] As described in our previous analysis,[[4](#_ENREF_4)] Dr. Angulo provided us with the raw data from his landmark study in order to determine the rate of indeterminate results given the prevalence of simple steatosis and NASH without advanced fibrosis in our study. These results are provided in Table 1.

**Liver Biopsy**

In this model, patients with NASH without advanced fibrosis who have false positive findings of advanced fibrosis on liver biopsy experience two costs associated with false positive results: the cost of annual specialist follow up and cirrhosis care as well as the lost effect of Vitamin E therapy. Similarly, patients with NASH who have false negative results of simple steatosis are not treated with Vitamin E. Patients with advanced fibrosis who have false negative results of NASH are treated with vitamin E but receive no benefit. The test characteristics of liver biopsy after a non-invasive test for fibrosis are unknown. Accordingly, for the strategies in our model that employ liver biopsy for patients with indeterminate results, it was assumed that biopsy test characteristics after non-invasive tests were perfect. The liver biopsy mortality rate is assumed to be 0.14%.[[5](#_ENREF_5)] The cost of biopsy is $1,558 (1168 - 1948) USD (2014).[[6](#_ENREF_6)] The cost of a fatal complication from liver biopsy is estimated to be $146,223 (2014 USD).[[7](#_ENREF_7)]

**Transition probabilities**

The transition probabilities are detailed in Supplementary Table 1. Ranges were derived from the references listed. When high quality data from population-wide sources were available, beta distributions were used.[[8](#_ENREF_8)] Beta distributions provide a probability density function between 0 and 1 with a parabola shaped by the probability given by the estimate. When drawn from beta distributions, the ranges listed reflect + two standard deviations. When single center estimates or author assumptions were available, a triangular distribution was employed reflecting the published range or a range of + 20% when one estimate was available.

The annual mortality rate was abstracted from the Center for Disease Control population-based life table and converted to a rate.[[9](#_ENREF_9)] Thereafter it was multiplied by the standardized mortality ratio for a patient with NAFLD/NASH - 1.34 (95% CI: 1.003–1.76) - and converted back to an annual probability of mortality for patients with NAFLD and NASH.[[10](#_ENREF_10)] Survival for patients with advanced fibrosis and compensated cirrhosis treated in a similar fashion, by adjusting CDC data with a mortality hazard ratio derived from a defined cohort of patients with NAFLD and advanced fibrosis or compensated cirrhosis - 3.28 (95% CI 2.27-4.76).[[11](#_ENREF_11)] Mortality rates for decompensated cirrhosis[[12](#_ENREF_12),[13](#_ENREF_13)], hepatocellular carcinoma[[14](#_ENREF_14)] and post-transplantation[[15](#_ENREF_15),[16](#_ENREF_16)] were independent of CDC data and abstracted from the relevant literature as listed in Supplementary Table 1.

Given the wide variability of treatment options for hepatocellular carcinoma, this model used generalizable data abstracted from actual care on a population level as recorded by the Surveillance, Epidemiology, and End Results (SEER) database. Using SEER, transition probabilities, treatment decisions and costs are divided by the stage of disease into nationally representative per-patient averages. In our model, the major branch points for state-transition were transplantation, resection, chemotherapy and palliative care. The transitions are not exclusive of local therapy such as transarterial chemoembolization or radiofrequency ablation which are utilized for each stage and contribute to the costs recorded in SEER. The model assumed that patients receiving chemotherapy would receive sorafenib which has emerged as the standard of care for chemotherapy candidates.

Patients were considered candidates for transplantation until age 65. Candidates for transplantation had decompensated cirrhosis and/or hepatocellular carcinoma. Once a patient under the age of 65 developed such an indication, they were considered for the transplant wait-list. The rate of rejection from the waitlist for patients with NAFLD has been assessed once previously at a large transplant center. This data was incorporated in a beta-distribution: 47.6 % (196/412).[[17](#_ENREF_17)]

**Costs**

Costs are detailed in supplementary table 2. This model was analyzed from the perspective of the healthcare system, accounting for direct medical costs alone. Gamma distributions were used for costs because during sensitivity analyses, costs cannot be less than 0 and exhibit a right skew (as often does cost data). Data was preferentially abstracted from primary studies of American healthcare costs, excluding reports of charges. Population-based averages were utilized for healthcare expenditures at each stage from routine care (for NAFLD, NASH and NASH with advanced fibrosis) to cirrhosis care (e.g. screening tests), stage-specific HCC care, and transplantation. However, where appropriate, additional one-time costs atop routine care (e.g. liver biopsy, specialist visit, medication) supplemented the otherwise average costs. When patients progressed to a more costly state in a given stage, a one-time transition cost equivalent to the differences in costs between states was assessed. When American data was unavailable, as in the case of VCTE,[[18](#_ENREF_18)] costs inflated to 2014 dollars in their original currency and then converted to American dollars using the appropriate conversion rates on August 16, 2014. All costs were rounded to the nearest dollar.

**Utilities**

Utilities are detailed in supplementary Table 3. Triangular distributions were employed for utilities with multiple estimates in the literature; otherwise for values without published ranges, a distribution of + 20% was assumed. Only patient-derived state-utility estimates were only included. The utility state associated with NAFLD (bland steatosis) was assumed to be equivalent to the well-state (1.0) without a range. Post-transplant utility was divided into year one and > year 2. It was assumed to be constant for all years after 2.[[19-21](#_ENREF_19)] When a patient transitioned to a state with lower utility during a given stage, a one-time disutility equivalent to the difference between states was assessed. Liver biopsy was associated with a one-time disutility of 0.005 QALY.[[22](#_ENREF_22)] Added pill burden from vitamin E and specialist visits were associated with a marginal disutility of 0.001 QALY.[[23](#_ENREF_23)]

**Data analysis**

For the purpose of a population EVPI calculation we determined likely number of potential subjects for which this model would apply. First, we determined the prevalence of NASH with reference to population data from the US census bureau. In 2013, there were a total of 4,511,845 50 year-old Americans. Though 46% of the population is felt to have NAFLD, the proportion of Americans with NASH (12% or 541,421) was employed for this analysis as these patients are most likely to be detected through liver enzyme evaluations.[[24](#_ENREF_24)] Second, we assume that the effective lifetime of a technology used for this purpose will be10 years. Thirds, the annual population EVPI was discounted at a rate of 3%.

**References**

1. Petta S, Di Marco V, Cammà C, Butera G, Cabibi D, Craxi A (2011) Reliability of liver stiffness measurement in non‐alcoholic fatty liver disease: the effects of body mass index. Alimentary pharmacology & therapeutics 33: 1350-1360.

2. Tsochatzis EA, Crossan C, Longworth L, Gurusamy K, Rodriguez-Peralvarez M, Mantzoukis K, et al. (2014) Cost-effectiveness of noninvasive liver fibrosis tests for treatment decisions in patients with chronic hepatitis C. Hepatology 60: 832-843.

3. Angulo P, Hui JM, Marchesini G, Bugianesi E, George J, Farrell GC, et al. (2007) The NAFLD fibrosis score: a noninvasive system that identifies liver fibrosis in patients with NAFLD. Hepatology 45: 846-854.

4. Tapper EB, Sengupta N, Hunink MM, Afdhal NH, Lai M (2015) Cost-Effective Evaluation of Nonalcoholic Fatty Liver Disease With NAFLD Fibrosis Score and Vibration Controlled Transient Elastography. The American journal of gastroenterology 110: 1298-1304.

5. Rockey DC, Caldwell SH, Goodman ZD, Nelson RC, Smith AD (2009) Liver biopsy. Hepatology 49: 1017-1044.

6. Hagan L, Yang Z, Ehteshami M, Schinazi R (2013) All‐oral, interferon‐free treatment for chronic hepatitis C: cost‐effectiveness analyses. Journal of viral hepatitis 20: 847-857.

7. Pasha T, Gabriel S, Therneau T, Dickson ER, Lindor KD (1998) Cost‐effectiveness of ultrasound‐guided liver biopsy. Hepatology 27: 1220-1226.

8. Hunink MGM WM, Wittenberg E, Drummond MF, Pliskin JS, Wong JB, Glasziou PP (2014) Decision making in health and medicine: Integrating evidence and values. Cambridge, UK: Cambridge University Press.

9. (2007) National Vital Statistics Reports. United States Life Tables. .

10. Adams LA, Lymp JF, St Sauver J, Sanderson SO, Lindor KD, Feldstein A, et al. (2005) The natural history of nonalcoholic fatty liver disease: a population-based cohort study. Gastroenterology 129: 113-121.

11. Ekstedt M, Hagström H, Nasr P, Fredrikson M, Stål P, Kechagias S, et al. (2015) Fibrosis stage is the strongest predictor for disease‐specific mortality in NAFLD after up to 33 years of follow‐up. Hepatology 61: 1547-1554.

12. Fleming KM, Aithal GP, Card TR, West J (2012) All‐cause mortality in people with cirrhosis compared with the general population: a population‐based cohort study. Liver International 32: 79-84.

13. Ratib S, Fleming KM, Crooks CJ, Aithal GP, West J (2014) 1 and 5 year survival estimates for people with cirrhosis of the liver in England, 1998–2009: A large population study. Journal of hepatology 60: 282-289.

14. Altekruse SF, McGlynn KA, Reichman ME (2009) Hepatocellular carcinoma incidence, mortality, and survival trends in the United States from 1975 to 2005. Journal of Clinical Oncology 27: 1485-1491.

15. Organ Procurement and Transplantation Network.

16. <http://srtr.org/annual_Reports/2011/data_tables_section9.aspx>.

17. O'Leary JG, Landaverde C, Jennings L, Goldstein RM, Davis GL (2011) Patients with NASH and cryptogenic cirrhosis are less likely than those with hepatitis C to receive liver transplants. Clinical Gastroenterology and Hepatology 9: 700-704. e701.

18. Steadman R, Myers RP, Leggett L, Lorenzetti D, Noseworthy T, Rose S, et al. (2013) A health technology assessment of transient elastography in adult liver disease. Canadian Journal of Gastroenterology 27: 149.

19. Siebert U, Sroczynski G, Rossol S, Wasem J, Ravens-Sieberer U, Kurth B, et al. (2003) Cost effectiveness of peginterferon α-2b plus ribavirin versus interferon α-2b plus ribavirin for initial treatment of chronic hepatitis C. Gut 52: 425-432.

20. McLernon DJ, Dillon J, Donnan PT (2008) Health-state utilities in liver disease: a systematic review. Medical Decision Making 28: 582-592.

21. Mahady SE, Wong G, Craig JC, George J (2012) Pioglitazone and vitamin E for nonalcoholic steatohepatitis: a cost utility analysis. Hepatology 56: 2172-2179.

22. Canavan C, Eisenburg J, Meng L, Corey K, Hur C (2013) Ultrasound elastography for fibrosis surveillance is cost effective in patients with chronic hepatitis C virus in the UK. Digestive diseases and sciences 58: 2691-2704.

23. Vijan S, Sussman JB, Yudkin JS, Hayward RA (2014) Effect of patients’ risks and references on health gains with plasma glucose level lowering in type 2 diabetes mellitus. JAMA Internal Medicine 174: 1227-1234.

24. Williams CD, Stengel J, Asike MI, Torres DM, Shaw J, Contreras M, et al. (2011) Prevalence of nonalcoholic fatty liver disease and nonalcoholic steatohepatitis among a largely middle-aged population utilizing ultrasound and liver biopsy: a prospective study. Gastroenterology 140: 124-131.

25. Wong VW-S, Wong GL-H, Choi PC-L, Chan AW-H, Li MK-P, Chan H-Y, et al. (2010) Disease progression of non-alcoholic fatty liver disease: a prospective study with paired liver biopsies at 3 years. Gut 59: 969-974.

26. Singh S, Allen AM, Wang Z, Prokop LJ, Murad MH, Loomba R (2015) Fibrosis progression in nonalcoholic fatty liver vs nonalcoholic steatohepatitis: a systematic review and meta-analysis of paired-biopsy studies. Clinical Gastroenterology and Hepatology 13: 643-654. e649.

27. Ekstedt M, Franzén LE, Mathiesen UL, Thorelius L, Holmqvist M, Bodemar G, et al. (2006) Long‐term follow‐up of patients with NAFLD and elevated liver enzymes. Hepatology 44: 865-873.

28. Adams LA, Sanderson S, Lindor KD, Angulo P (2005) The histological course of nonalcoholic fatty liver disease: a longitudinal study of 103 patients with sequential liver biopsies. Journal of hepatology 42: 132-138.

29. Sanyal AJ, Chalasani N, Kowdley KV, McCullough A, Diehl AM, Bass NM, et al. (2010) Pioglitazone, vitamin E, or placebo for nonalcoholic steatohepatitis. New England Journal of Medicine 362: 1675-1685.

30. Fleming KM, Aithal G, Card T, West J (2010) The rate of decompensation and clinical progression of disease in people with cirrhosis: a cohort study. Alimentary pharmacology & therapeutics 32: 1343-1350.

31. Ascha MS, Hanouneh IA, Lopez R, Tamimi TAR, Feldstein AF, Zein NN (2010) The incidence and risk factors of hepatocellular carcinoma in patients with nonalcoholic steatohepatitis. Hepatology 51: 1972-1978.

32. Sanyal AJ, Banas C, Sargeant C, Luketic VA, Sterling RK, Stravitz RT, et al. (2006) Similarities and differences in outcomes of cirrhosis due to nonalcoholic steatohepatitis and hepatitis C. Hepatology 43: 682-689.

33. Jain A, Reyes J, Kashyap R, Dodson SF, Demetris AJ, Ruppert K, et al. (2000) Long-term survival after liver transplantation in 4,000 consecutive patients at a single center. Annals of surgery 232: 490.

34. Lang K, Danchenko N, Gondek K, Shah S, Thompson D (2009) The burden of illness associated with hepatocellular carcinoma in the United States. Journal of hepatology 50: 89-99.

35. Llovet JM, Ricci S, Mazzaferro V, Hilgard P, Gane E, Blanc J-F, et al. (2008) Sorafenib in advanced hepatocellular carcinoma. New England Journal of Medicine 359: 378-390.

36. Bennett WG, Inoue Y, Beck JR, Wong JB, Pauker SG, Davis GL (1997) Estimates of the cost-effectiveness of a single course of interferon-α2b in patients with histologically mild chronic hepatitis C. Annals of internal medicine 127: 855-865.

37. Coffin PO, Scott JD, Golden MR, Sullivan SD (2012) Cost-effectiveness and population outcomes of general population screening for hepatitis C. Clinical infectious diseases 54: 1259-1271.

38. (2012) Kaiser Permanente Chargemaster. Charge Master.

39. Davey PJ, Schulz M, Gliksman M, Dobson M, Aristides M, Stephens NG (1998) Cost–effectiveness of vitamin E therapy in the treatment of patients with angiographically proven coronary narrowing (CHAOS trial). The American journal of cardiology 82: 414-417.

40. Eddy DM, Schlessinger L, Kahn R (2005) Clinical outcomes and cost-effectiveness of strategies for managing people at high risk for diabetes. Annals of Internal medicine 143: 251-264.

41. Group DPPR (2003) Costs associated with the primary prevention of type 2 diabetes mellitus in the diabetes prevention program. Diabetes Care 26: 36-47.

42. Thein HH, Isaranuwatchai W, Campitelli MA, Feld JJ, Yoshida E, Sherman M, et al. (2013) Health care costs associated with hepatocellular carcinoma: A population‐based study. Hepatology 58: 1375-1384.

43. Tan JA, Joseph TA, Saab S (2008) Treating hepatitis C in the prison population is cost‐saving. Hepatology 48: 1387-1395.

44. Lin O, Keeffe E, Sanders G, Owens D (2004) Cost‐effectiveness of screening for hepatocellular carcinoma in patients with cirrhosis due to chronic hepatitis C. Alimentary pharmacology & therapeutics 19: 1159-1172.

45. McCusker J, Brudevold C (1984) Health services utilization and costs of care in terminal cancer: implications for hospice programs. Home health care services quarterly 5: 61-74.

46. Polednak A, Shevchenko I (1997) Hospital charges for terminal care of cancer patients dying before age 65. Journal of health care finance 25: 26-34.

47. Showstack J, Katz PP, Lake JR, Brown Jr RS, Dudley RA, Belle S, et al. (1999) Resource utilization in liver transplantation: effects of patient characteristics and clinical practice. JAMA 281: 1381-1386.

48. Hogan C, Lunney J, Gabel J, Lynn J (2001) Medicare beneficiaries’ costs of care in the last year of life. Health affairs 20: 188-195.

49. Hoover DR, Crystal S, Kumar R, Sambamoorthi U, Cantor JC (2002) Medical expenditures during the last year of life: findings from the 1992–1996 Medicare current beneficiary survey. Health services research 37: 1625-1642.

50. Dan AA, Kallman JB, Srivastava R, Younoszai Z, Kim A, Younossi ZM (2008) Impact of chronic liver disease and cirrhosis on health utilities using SF‐6D and the health utility index. Liver Transplantation 14: 321-326.

51. Younossi ZM, Boparai N, McCormick M, Price LL, Guyatt G (2001) Assessment of utilities and health-related quality of life in patients with chronic liver disease. The American journal of gastroenterology 96: 579-583.

52. Chong CA, Gulamhussein A, Heathcote EJ, Lilly L, Sherman M, Naglie G, et al. (2003) Health-state utilities and quality of life in hepatitis C patients. The American journal of gastroenterology 98: 630-638.

53. Thein H-H, Krahn M, Kaldor JM, Dore GJ (2005) Estimation of utilities for chronic hepatitis C from SF-36 scores. The American journal of gastroenterology 100: 643-651.

54. Ratcliffe J, Longworth L, Young T, Bryan S, Burroughs A, Buxton M (2002) Assessing health‐related quality of life pre–and post–liver transplantation: A prospective multicenter study. Liver Transplantation 8: 263-270.

55. Cucchetti A, Piscaglia F, Cescon M, Colecchia A, Ercolani G, Bolondi L, et al. (2013) Cost-effectiveness of hepatic resection versus percutaneous radiofrequency ablation for early hepatocellular carcinoma. Journal of hepatology 59: 300-307.

**Supplementary Table 1: Reference case estimates and distributions of probabilities used in the model[**[**4**](#_ENREF_4)**]**

| **Health State** | **Estimate (Distribution)** | **Reference** |
| --- | --- | --- |
| Probability of NAFLD developing NASH | 0.028 (0.00 – 0.063) | [[25](#_ENREF_25)] |
| Probability of NAFLD progressing by a fibrosis stage | 0.07 (0.02 – 0.11) | [[26](#_ENREF_26)] |
| Probability of NASH progressing by a fibrosis stage | 0.14 (0.07 – 0.21) | [[26](#_ENREF_26)] |
| Probability of advanced fibrosis progressing to cirrhosis* | 0.072 (0.057 - 0.086) | [[27](#_ENREF_27)] |
| Probability of NASH regressing to NAFLD | 0.038 (0.00 – 0.09) | [[28](#_ENREF_28)] |
| Probability of advanced fibrosis regressing to NASH | 0.029 (0.00 – 0.09) | [[28](#_ENREF_28),[29](#_ENREF_29)] |
| **Cirrhosis** |  |  |
| Probability of decompensation |  |  |
| During first year of diagnosis | 0.25 (0.23 - 0.28) | [[30](#_ENREF_30)] |
| After first year of diagnosis | 0.055 (0.048 - 0.062) | [[30](#_ENREF_30)] |
| Probability of developing hepatocellular carcinoma | 0.026 (0.026 - 0.05) | [[31](#_ENREF_31),[32](#_ENREF_32)] |
| **Decompensated Cirrhosis** |  |  |
| Probability of developing hepatocellular carcinoma | 0.026 (0.026 - 0.05) | [[31](#_ENREF_31),[32](#_ENREF_32)] |
| Probability of liver transplant for listed patients | 0.34 (0.32 – 0.37) | [[16](#_ENREF_16)] |
| Probability of all cause mortality | 0.16 (0.15-0.38) | [[12](#_ENREF_12),[13](#_ENREF_13)] |
| **Post liver transplant** |  |  |
| Probability of survival during first year | 0.86 (0.86 – 0.87) | [[15](#_ENREF_15)] |
| Probability of survival after first year | 0.0.93 (0.92 – 0.95) | [[33](#_ENREF_33)] |
| **Hepatocellular Carcinoma (HCC)** |  |  |
| Localized Stage at diagnosis* | 0.57 (0.46 – 0.68) | [[34](#_ENREF_34)] |
| Transplant (Age < 65) | 0.04 (0.03 – 0.05) | [[34](#_ENREF_34)] |
| Resection (Age < 65) | 0.12 (0.11 – 0.14) | [[34](#_ENREF_34)] |
| Resection (Age > 65) | 0.10 (0.09 – 0.12) | [[34](#_ENREF_34)] |
| Mortality | 0.23 (0.20 – 0.26) | [[14](#_ENREF_14)] |
| Regional Stage at diagnosis |  |  |
| Resection (Age < 65) | 0.065 (0.05 – 0.08) | [[34](#_ENREF_34)] |
| Resection (Age > 65) | 0.0092 (0.005 – 0.014) | [[34](#_ENREF_34)] |
| Sorafenib | 0.15 (0.14 – 0.16) | [[34](#_ENREF_34)] |
| Mortality | 0.21 (0.18 – 0.24) | [[14](#_ENREF_14)] |
| Distant Stage at diagnosis* | 0.19 (0.15 – 0.22) | [[34](#_ENREF_34)] |
| Sorafenib | 0.25 (0.20 – 0.30) | [[34](#_ENREF_34)] |
| Mortality during first year of sorafenib | 0.56 (0.50 - 0.62) | [[35](#_ENREF_35)] |
| Transition to palliative care from sorafenib | 0.9 | [assumption](#_ENREF_31) |
| Mortality after first year of sorafenib | 0.85 | [[35](#_ENREF_35)] / assumption |
| Mortality during palliative care | 0.94 (0.92 – 0.96) | [[14](#_ENREF_14)] |

NAFLD = Nonalcoholic Fatty Liver Disease , NASH = Nonalcoholic Steatohepatitis
All estimates are assessed in the probabilistic decision model using beta distributions except where an (*) indicates a triangular distribution.

| **Supplementary Table 2: Estimated Costs**  **in 2014 US dollars** |  |  |  |  |
| --- | --- | --- | --- | --- |
|  | Estimate | Range | Reference | |
| **Annual Costs** |  |  |  | |
| Routine specialist care  (no therapy) | 244 | 90 – 537 | [[36](#_ENREF_36)] | |
| Compensated Cirrhosis | 1,268 | 742 – 1,793 | [[6](#_ENREF_6),[36](#_ENREF_36),[37](#_ENREF_37)] | |
| Decompensated cirrhosis | 16,263 | 13,011 – 40,198 | [[6](#_ENREF_6),[36](#_ENREF_36),[37](#_ENREF_37)] | |
| Specialist Annual Visit | 249 | 199 – 299 | [[38](#_ENREF_38)] | |
| Vitamin E | 70 | 70 – 164 | [[39](#_ENREF_39)] | |
| Lifestyle modifications | 1,877 | 1,502 – 2,252 | [[40](#_ENREF_40),[41](#_ENREF_41)] | |
| **Hepatocellular Carcinoma** |  |  |  | |
| First year of diagnosis | 41,460 | 29,141 – 51592 | [[37](#_ENREF_37),[42](#_ENREF_42),[43](#_ENREF_43)] | |
| Localized | 42,645 | 38,380 – 46,910 | [[37](#_ENREF_37),[42](#_ENREF_42),[43](#_ENREF_43)] | |
| Regional | 39,421 | 35,479 – 43,363 | [[37](#_ENREF_37),[42](#_ENREF_42),[43](#_ENREF_43)] | |
| Distant | 33,064 | 29,758 – 41,580 | [[37](#_ENREF_37),[42](#_ENREF_42),[43](#_ENREF_43)] | |
| Distant - Sorafenib | 80,117 | 64,094 – 96,141 | [[21](#_ENREF_21)] | |
| Palliative care | 44,042 | 22,021 – 88,083 | [[44-46](#_ENREF_44)] | |
| **One Time Costs** |  |  |  | |
| Vibration-Controlled Transient-Elastography | 100 | 80 – 120 | [[18](#_ENREF_18)] | |
| Liver Biopsy | 1,558 | 1,168 – 1,948 | [[6](#_ENREF_6)] | |
| Liver resection | 40,156 | 20,078 – 80,311 | [[44-46](#_ENREF_44)] | |
| Liver Transplant (First Year) | 318,157 | 247,679 – 318,157 | [[34](#_ENREF_34),[47](#_ENREF_47)] | |
| Death from any cause | 57,088 | 35,987 – 61,088 | [[48](#_ENREF_48),[49](#_ENREF_49)] | |

All costs are assessed in the probabilistic decision model using gamma distributions. All costs were used in the previously published microsimulation model.[[4](#_ENREF_4)]

| Supplementary Table 3: Health State quality of life weights |  | |  |  |  |
| --- | --- | --- | --- | --- | --- |
| Health State | Estimate | | Range | | Reference |
| Nonalcoholic Fatty Liver Disease | 1 | * | |  | assumption |
| Nonalcoholic Steatohepatitis | 0.95 | 0.90 – 1.0 | | [[19](#_ENREF_19)] | |
| Well with Advanced Fibrosis | 0.92 | 0.65 – 0.95 | | [[50](#_ENREF_50),[51](#_ENREF_51)] | |
| Compensated cirrhosis | 0.78 | 0.71 – 0.89 | | [[19](#_ENREF_19),[50-52](#_ENREF_50)] | |
| Decompensated cirrhosis | 0.6 | 0.46 – 0.71 | | [[50-54](#_ENREF_50)] | |
| Liver transplant (1st year) | 0.69 | 0.55 – 0.78 | | [[54](#_ENREF_54)] | |
| Liver transplant (2nd year) | 0.79 | 0.62 – 0.79 | | [[19](#_ENREF_19),[52](#_ENREF_52),[54](#_ENREF_54)] | |
| Hepatocellular Carcinoma (HCC) | 0.65 | 0.52 – 0.78 | | [[52](#_ENREF_52)] | |
| Decompensated cirrhosis and HCC | 0.57 | 0.46 – 0.68 | | [[55](#_ENREF_55)] | |
| Palliative care | 0.40 | 0.32 – 0.48 | | [[55](#_ENREF_55)] | |

All estimates are assessed in the probabilistic decision model using triangular distributions. All utilities were used in the previously published microsimulation model.[[4](#_ENREF_4)]
